# Peri-implantitis: a systemic burden for our patients

**DOI:** 10.3389/fdmed.2026.1774378

**Published:** 2026-03-06

**Authors:** Emilio A. Cafferata, Frank Schwarz

**Affiliations:** 1 Oral Peri-Implant Research Group, Department of Oral Implantology, Postgraduate School of Dentistry, Universidad Científica del Sur, Lima, Perú; 2 Department of Oral Surgery, Implantology and Oral Medicine, Goethe Universität, Frankfurt am Main, Germany

**Keywords:** chronic inflammation, inflammation, inflammatory mediators, peri-implant disease, systemic disease, systemic inflammation

## Abstract

Peri-implantitis—an increasingly prevalent chronic inflammatory disease—has been linked not only to peri-implant bone destruction and implant failure, but also to systemic inflammation and chronic inflammatory diseases. Accumulating evidence suggest that peri-implant inflammation can contribute to a sustained systemic low-grade inflammatory state characterized by elevated inflammatory mediators, like interleukin (IL)-6 and C reactive protein, episodic bacteremia, and metabolic alterations. This persistent inflammatory burden has been associated with distal organ dysfunction, including cardiovascular and renal complications, and neuroinflammatory processes implicated in cognitive decline and neurodegeneration. In parallel, implant-supported rehabilitation and long-term maintenance has been linked to surrogate markers of cognitive resilience, whereas treatment of peri-implant diseases aimed at reducing local inflammation may, at the same, be associated with the improvement of systemic inflammatory markers. This concise review synthesizes mechanistic and clinical evidence that peri-implantitis is not only an oral disease but also a systemic health modifier, highlights current knowledge gaps, and outlines priorities for research and clinical practice.

## 1 Introduction

Dental implants are widely considered the first choice to restore oral function in adult patients, yet peri-implant diseases—i.e., peri-implant mucositis and peri-implantitis (PI)—affect a substantial proportion of implant recipients (1). PI is one of the leading causes of implant loss—leading to impaired mastication, speech, aesthetics and overall quality of life (2, 3). Arising from dysbiosis coupled with chronic deregulated inflammation, the progressive destruction of peri-implant tissues during PI not only jeopardizes implant survival but may also have systemic consequences.

While a robust body of literature links periodontitis-associated dysbiosis and deregulated inflammation to multiple systemic diseases—including cardiovascular disorders, diabetes, rheumatoid arthritis, chronic kidney disease, and neurodegenerative conditions (4), evidence supporting similar systemic associations for PI remains limited and still under development. Even though periodontitis and PI share etiological factors and overlapping clinical features, they are biologically and clinically distinct entities, differing in tissue architecture, immune response, and disease progression. Consequently, findings derived from periodontitis cannot be assumed to directly translate to PI, and evidence supporting similarity between their systemic associations remains comparatively limited.

Nevertheless, oral inflammatory conditions, such as PI, are considered persistent sources of inflammatory mediators, such as pro-inflammatory cytokines and damage-associated molecular patterns, with the potential to disseminate into systemic circulation. In addition, bacteria, their metabolites, and pathogen-associated molecular patterns, can translocate beyond the oral cavity, further sustaining systemic low-grade inflammation and contributing to distal organ dysfunction (4–6). However, evidence for PI remains largely extrapolated from experimental models and indirect clinical observations thus inflammatory pathways remain leading mechanistic hypotheses.

Given the globally alarming prevalence of PI, revising its potential role as a systemic inflammatory modifier is becoming increasingly fundamental. Therefore, this review synthesizes the current PI-specific mechanistic and clinical evidence suggesting PI could be associated to systemic inflammation and chronic inflammatory diseases, and may participate in the pathogenesis or progression of distal organ impairment, including neuroinflammatory and metabolic pathways linked to cognitive decline.

## 2 Implant-peri-implant mucosa barrier breach

Peri-implant tissues appear to be more susceptible to attachment loss and persistent biofilm accumulation than periodontal tissues due to their unique structural, immunological, and microbiological characteristics (7). The absence of a robust connective tissue attachment, characterized by collagen fibers running parallel to the implant surface rather than inserting perpendicularly as in natural teeth, results in a weaker soft tissue seal and reduced capacity for immune surveillance and repair. Consequently, peri-implant tissues are more prone to rapid and extensive tissue destruction when exposed to biofilm-induced inflammation, making this breakdown less effectively contained than in periodontal tissue (8).

Compromised mucosal integrity and ulcerated peri-implant pockets—marked by loss of junctional proteins, increased intercellular edema, and inflammatory infiltration—lead to greater epithelial permeability facilitating the possibility of episodic bacteremia and dissemination of microbial components (9). Although this breakdown provides a biologically plausible pathway for microbial translocation, direct bacteremia attributable to PI has not yet been demonstrated in clinical studies, highlighting a critical evidence gap. Nevertheless, the systemic impact of peri-implant dysbiosis supports that translocation of microbial byproducts into the circulation is possible, as a consequence of an impaired oral mucosal barrier and contributing to systemic inflammation (8, 10).

## 3 Chronic release of pro-inflammatory mediators

As peri-implant mucosal integrity deteriorates, the inflamed peri-implant tissues become a continuous source of pro-inflammatory mediators with effects extending beyond the oral cavity. Indeed, the sustained release of pro-inflammatory cytokines and acute-phase reactants from PI lesions—such as IL-6, tumor necrosis factor (TNF)-*α*, and CRP—into the systemic circulation contributes to a state of chronic low-grade inflammation which is strongly associated with endothelial dysfunction, distal tissue injury, and the progression of systemic diseases (11, 12).

Consistent clinical evidence indicates that patients with PI exhibit markedly elevated levels of circulating pro-inflammatory markers, including up to threefold higher CRP concentrations, and nearly doubled IL-6 levels compared with healthy implant controls (13–16). Notably, CRP concentrations have been shown to increase in a stepwise manner from peri-implant health to peri-implant mucositis, and reach their highest levels in established PI (15, 17). Reinforcing these observations, a systematic review and meta-analysis demonstrated that PI is associated with elevated systemic inflammatory markers—most consistently CRP, IL-6, and leukocyte counts—relative to healthy individuals (18). Importantly, these same inflammatory mediators are well-established predictors of adverse systemic outcomes, as elevated CRP and IL-6 levels are associated with higher mortality rates, greater organ dysfunction, and an increased need for organ support, such as vasopressors and renal replacement therapy, in critically ill patients (18, 19).

Apart from IL-6 and CRP, increased serum levels of pro-inflammatory cytokines—including IL-1β, IL-17A and TNF-α—have also been detected in patients with PI, while showing significant correlations with clinical indicators of disease severity (20, 21). Notably, synergistic interactions among IL-1β, TNF-α, and IL-17 are known to amplify inflammatory responses in autoimmune and chronic inflammatory diseases, driving organ-specific damage, accelerating disease progression, and increasing morbidity (22). Taken together, these observations suggest that as PI progresses, the escalating local inflammatory burden may parallel and potentially contribute to heightened systemic inflammation, underscoring the capacity of PI–derived inflammatory mediators to exert biological effects beyond the oral environment, with possible implications for distant organs such as the liver, cardiovascular system, and central nervous system.

## 4 Dissemination of microbial byproducts during peri-implantitis

Beyond local tissue destruction, PI may contribute to systemic inflammatory burden by facilitating episodic bacteremia and the potential release of microbial components into systemic circulation. Although direct evidence linking peri-implant bacteremia to specific systemic outcomes remains limited, mechanistic studies support the possibility of increased epithelial permeability and microbial translocation during PI. Supporting this concept, a recent case reported a patient with multiple implants affected by PI and concurrent periodontitis who developed a paravertebral abscess caused by *Streptococcus parasanguinis*, an oral commensal with opportunistic pathogenic potential. Successful treatment required combined implant and tooth extraction along with systemic antibiotic therapy. Interestingly, bacterial loads recovered from PI-affected implants exceeded those found on teeth, suggesting that implants may act as significant reservoirs for systemic bacterial dissemination (23).

## 5 Systemic consequences of peri-implantitis

### 5.1 Metabolic disturbance

Emerging evidence suggests that PI may extend its impact beyond local and systemic inflammatory dysregulation, potentially contributing to broader metabolic alterations. Observational studies have linked PI with dyslipidemia, hyperglycemia and markers of renal dysfunction, mirroring findings from periodontitis, where periodontal therapy results in clinically meaningful reductions in glucose or insulin levels—sometimes even comparable to lifestyle interventions magnitude—supporting a biologically relevant systemic effect of PI-derived inflammation.

Patients affected by PI often display signs of dyslipidemia, characterized by increased total cholesterol and low-density lipoprotein (LDL) levels, accompanied by elevated systemic TNF-α and reduced anti-inflammatory IL-10 concentrations. These systemic lipid abnormalities also show significant correlations with probing depth (PD) and bleeding on probing (BOP) (24). Similarly, in a large retrospective cohort study of 621 patients, both fasting glucose and HbA1c levels were significantly associated with the severity of bone loss (*p* < 0.01). Supporting these findings, a meta-analysis confirmed that PI clinical progression indices—including marginal bone loss, PD, BOP, and plaque index—are significantly higher in patients with diabetes compared to non-diabetic patients, even over short follow-up periods (25). Moreover, markers of renal dysfunction, including blood urea nitrogen and creatinine levels, were significantly associated to PI (*p* < 0.05), and multilevel regression analysis identifying blood urea nitrogen as an independent predictor of peri-implant bone loss [OR = 1.082, CI (1.027–1.141), *p* = 0.003] (26). Collectively, these observational data suggest that PI may not only contribute to systemic inflammation but also be associated with metabolic alterations, potentially linking it, for instance, to cardiovascular and renal comorbidities,

### 5.2 Peri-implant health, neuroinflammation and cognitive decline

Growing recognition of the bidirectional relation between oral health and cognitive aging has significantly shifted the perception of oral diseases from merely local affections to potential contributors to neurocognitive decline. Epidemiological evidence have consistently reported associations between poor oral health and an increased risk of cognitive impairment, ranging from mild cognitive decline (27) to dementia (28). While cognitive deterioration itself may compromise oral self-care, aging-related frailty—including reduced manual dexterity, lower energy levels, and increase reliance on caregivers—may also play a critical role in declining oral hygiene among older adults (29).

Alongside aging and tooth loss, the frequency of dental implant therapy among older individuals continues to rise (30). Although approximately 20% of residents in nursing homes and caregiving facilities have dental implants, many caregivers lack the training required to identify implants or understand their specific maintenance needs, highlighting a critical gap in implant-related care for frail patients (31). Nevertheless, the restoration of masticatory function with implant-supported prostheses has been associated with the preservation of brain volume, enhanced neural activity, and better oral health-related quality of life for older patients, suggesting that this functional rehabilitation may indirectly support cognitive resilience.

In this context, direct clinical evidence linking peri-implant diseases to cognitive performance is currently limited. Inflammatory pathways represent a leading mechanistic hypothesis, possibly associating PI with neuroinflammation and neurodegeneration. A chronic PI model by our group showed that chronic peri-implant inflammation is associated to neuroinflammatory responses—such as microglial activation, increased detection of pro-inflammatory cytokines in the brain, and histological signs of neurodegeneration—providing a biologically plausible route from PI to neuroinflammatory and neurodegenerative changes (32). Despite the complexity of these interactions, the consistency of epidemiological and pre-clinical findings underscore the potential role of peri-implant health as potentially modifiable factors in cognitive aging.

Elevated circulating IL-6—consistently reported in PI—is a well-established biomarker of neuroinflammation and has been longitudinally associated with accelerated decline in executive function, reasoning, verbal fluency, and global cognition, as well as with structural brain changes including increased white matter hyperintensity, and reduced gray matter and hippocampal volume (33–35). In contrast, CRP shows weaker associations with cognition, although chronically elevated levels—also observed in PI—have been linked to subclinical cerebral small vessel disease and reduced brain volume, particularly in patients with amyloid-β pathology (36, 37). In parallel, dyslipidemia—as reported during PI—has been implicated in neurodegenerative processes. Elevated LDL-cholesterol, small dense LDL, and apolipoprotein B predict faster cognitive decline and increased Alzheimer's disease risk in longitudinal cohorts, are associated with greater amyloid and tau pathology in autopsy studies, and promote microglial activation and pro-inflammatory cytokine signaling in experimental models (38–41). Together, these data suggest that the systemic inflammatory and dyslipidemic milieu associated with PI—characterized by IL-6, CRP and lipid abnormalities—may provide an additional biologically plausible mechanistic bridge between chronic oral inflammation and neuroinflammatory processes underlying cognitive decline and neurodegeneration.

Nevertheless, the current state of the evidence linking peri-implant health to cognitive outcomes is predominantly observational and subject to residual confounding, possibly arising from age, frailty, inflammatory conditions, socioeconomic status, and even oral hygiene. Therefore, these findings should be interpreted cautiously and should not be used to infer a direct protective effect of peri-implant health on cognitive trajectories. While preclinical models support biological plausibility, well-designed longitudinal cohorts and interventional studies with appropriately reported control of confounders are required to determine whether peri-implant health represents a truly modifiable factor influencing cognitive aging.

### 5.3 Peri-implantitis and malignancies

As chronic inflammation facilitates the development of a pro-oncogenic microenvironment that sustains cancer progression, it has been questioned whether persistent peri-implant inflammation could have similar implications. An increasing number of case reports and retrospective studies have described malignant tumours developing in proximity to implants affected by PI, typically appearing within months to a median of five years following implant placement (43). However, these observations predominantly reflect a diagnostic overlap between peri-implant inflammatory lesions and early-stage oral squamous cell carcinoma rather than a possibly oncogenic effect of PI. Approximately 50% of peri-implant-adjacent malignant lesions are initially misdiagnosed as PI, with a final squamous cell carcinoma in up to 97% of cases, highlighting the critical importance of routine histopathological assessment—particularly for persistent or atypical lesions unresponsive to conventional therapy—even in patients without established oncologic risk factors (43).

Although chronic inflammation is recognized as a contributing factor in carcinogenesis across multiple tissues, prospective studies are required to determine whether peri-implant inflammation may have any independent role in oral malignant transformation (42). Therefore, the currently available evidence supports the importance of diagnostic vigilance rather than suggesting a causal oncogenic role of PI (44):

## 6 Patient awareness of peri-implantitis and its consequences

Despite the increasing prevalence of dental implants and peri-implant diseases, patient awareness of PI and its potential consequences remains generally low. Survey studies indicate that many implant recipients have limited knowledge of peri-implant diseases as distinct entities, their risk factors, and the importance of maintenance care (45), and may often perceive diseased implants as healthy, thereby underestimating the risk of chronic inflammation, bone loss, and implant failure (46).

Public and patient awareness of the systemic implications of PI remains similarly limited, leading many to underestimate its potential impact on overall health (47). Although most patients can recognize some clinical signs of periodontal disease, the majority of patients with dental implants do not recognize PI as a distinct pathology. For instance, 74.1% of patients reported lacking knowledge about PI (45), and nearly 89% of affected implants were perceived as “healthy” by their owners (46). Even patients who regularly attend dental or medical appointments, including those affected by associated comorbidities, demonstrate similarly low levels of awareness (48). Collectively, these findings highlight a significant gap in patients’ health literacy regarding the PI-systemic disease connection, emphasising the need to communicate the growing evidence linking it to systemic inflammation to patients effectively.

Therefore, this lack of awareness may hinder early detection and timely intervention of PI, particularly among older adults who are most vulnerable to systemic consequences of chronic oral inflammation, highlight the crucial importance of targeted patient and caregiver education and integrating implant maintenance into routine chronic disease monitoring programs to preserve both oral and systemic health.

## 7 Implant therapy and peri-implantitis treatment effect on systemic health

While untreated PI is significantly associated to sustained local and systemic inflammatory burden, effective implant rehabilitation and timely treatment of PI may reduce the inflammatory tone and improve patient-reported outcomes. On the one hand, implant therapy—by restoring and enhancing masticatory function—may improve nutritional status, and oral-health–related quality of life in older adults, with potential associated neurocognitive benefits (49). Partially dentate patients rehabilitated with implant- or mucosa-supported prostheses show higher baseline cognitive performance [*β* = 1.032 (95% CI: 0.813–1.251); *p* < 0.001] and a significantly slower trajectory of cognitive decline over time [*β* = 0.127 (95% CI: 0.047–0.206); *p* < 0.01] (50). Accordingly, the preservation of functional masticatory pairs has been linked with greater maintenance of brain volume, including white matter, grey matter and cortical thickness (51). In this context, improved oral function and masticatory efficiency with implant-supported prostheses have been linked to superior cognitive performance and enhanced brain activity compared with complete dentures (52), even among individuals with cognitive impairment and early dementia (53).

On the other hand, emerging clinical evidence indicates that non-surgical and surgical management of PI can potentially reduce systemic inflammatory markers, supporting the concept that controlling peri-implant inflammation confers measurable systemic benefits. A recent clinical trial demonstrated that PI treatment, with or without adjunctive systemic antibiotics, significantly reduced circulating levels of inflammatory mediators—including CRP, LDL cholesterol and TNF-*α*—6 months after therapy (54). In this line, treatment of experimental PI via open flap debridement resulted in normalization of blood leukocyte count and hematological parameters, as well as restoration of inflammatory albumin and hepatic damage aspartate aminotransferase markers levels to baseline in an animal model (55, 56).

Collectively, these findings suggest that integral implant-supported rehabilitation, combined with effective PI management, may not only restore oral function and reduce systemic inflammatory burden but also contribute to the maintenance of cognitive health in older adults. Nevertheless, further longitudinal and interventional studies are needed to determine the durability of these effects and their broader clinical implications.

## 8 Conclusion

**Figure 1:**
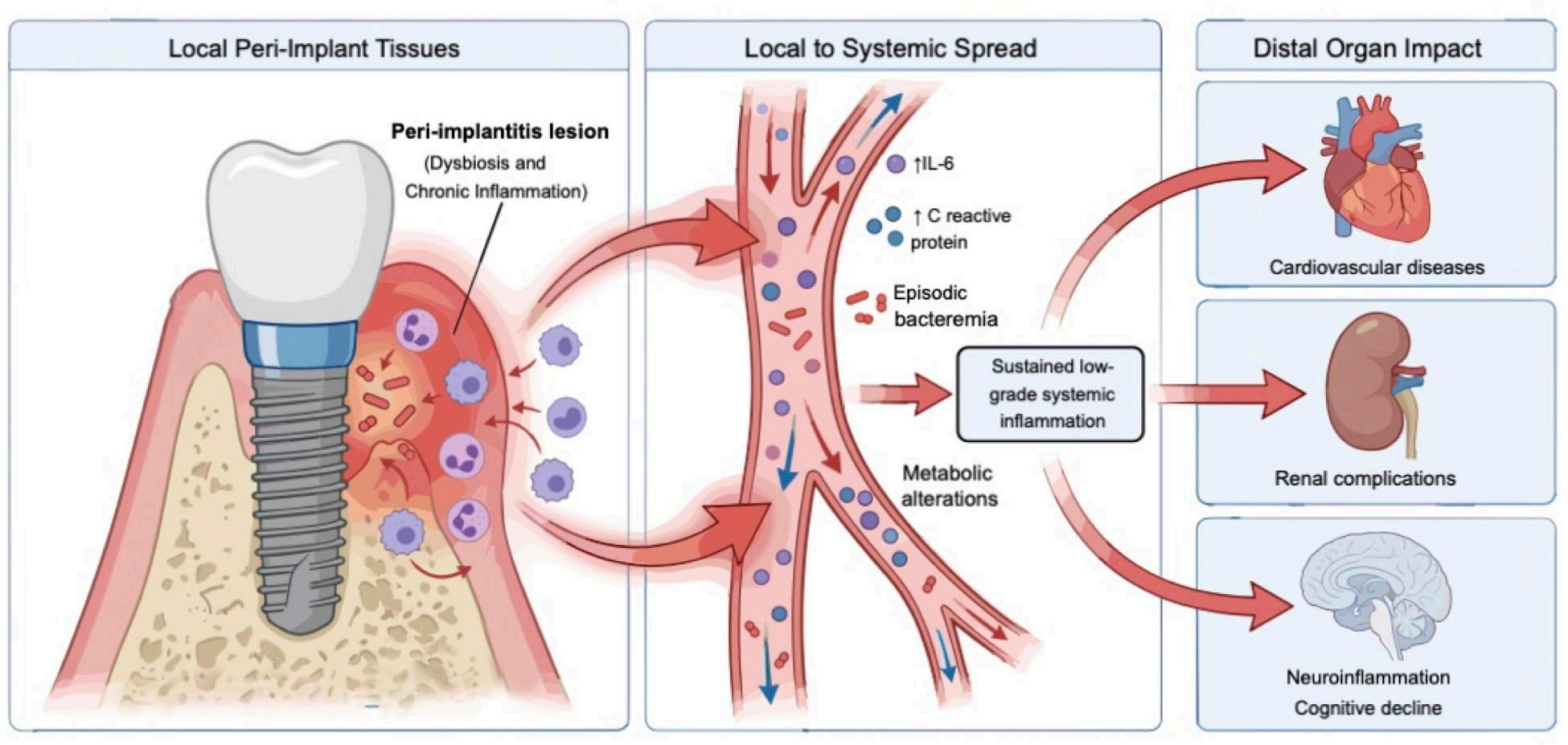
Graphical abstract illustrating the proposed mechanisms by which peri-implantitis may be associated with systemic inflammation and distal organ effects. Peri-implant microbiota dysbiosis and dysregulated chronic inflammation may facilitate the dissemination of inflammatory mediators into the circulation, potentially enhanced by ulceration or increased permeability of the peri-implant mucosa. This process has been associated with episodic bacteremia and elevated systemic levels of interleukin-6 and C-reactive protein, which together may relate to metabolic alterations and the establishment of a sustained low-grade inflammatory state. Such systemic changes have been implicated in cardiovascular and renal diseases, as well as neuroinflammatory processes associated with neurodegeneration and cognitive decline. The illustrated pathways represent biologically plausible mechanisms derived from observational and experimental evidence without inferring causality.

Peri-implant health should be considered as an integral component of overall health rather than an isolated local condition. PI is associated with sustained local inflammation and elevated systemic inflammatory markers, which may contribute to metabolic dysregulation and neuroinflammatory processes through mechanisms such as inflammatory mediator spillover and episodic bacteremia (Figure 1). Evidence indicates that structured peri-implant maintenance and timely treatment is associated with a potential reduction of the systemic inflammatory burden, improvement of metabolic profiles, and restoration of oral function, while implant-supported rehabilitation is associated with improved quality of life and more favorable cognitive outcomes. Although findings remain largely from associative and pre-clinical studies, these findings support the view that maintaining peri-implant health represents a potentially modifiable factor for mitigating systemic inflammatory burden and supporting healthy aging.
